# Hydrology and Ecological Evolution in a Permafrost Region in Northeast China Since the Late Pleistocene

**DOI:** 10.1002/ece3.71935

**Published:** 2025-09-12

**Authors:** Rui Liu, Lin Zhao, Xiaodong Wu, Xiaofeng Cheng, Boxiong Zhang, Jianxiang He, Dongyu Yang, Shuying Zang

**Affiliations:** ^1^ Heilongjiang Province Key Laboratory of Geographical Environment Monitoring and Spatial Information Service in Cold Regions Harbin Normal University Harbin China; ^2^ Heilongjiang Province Collaborative Innovation Center of Cold Region Ecological Safety Harbin China; ^3^ School of Geographical Sciences Nanjing University of Information Science and Technology Nanjing China; ^4^ Cryosphere Research Station on the Qinghai‐Tibet Plateau, State Key Laboratory of Cryospheric Science, Northwest Institute of Eco‐Environment and Resources Chinese Academy of Sciences Lanzhou China; ^5^ International Research Center for China‐Mongolia‐Russia Cold and Arid Regions Environment and Engineering, Northwest Instituteof Eco‐Environment and Resources Chinese Academy of Sciences Lanzhou China

**Keywords:** East Asian monsoon, Late Pleistocene, Mohe Basin, organic matter, paleoecology, permafrost effect

## Abstract

Northeast China is the southern margin of the Eurasian permafrost region, which is very sensitive to global change. However, the historical hydrology and ecological evolution in a concentrated distribution of permafrost in this region remain unclear, hindering our understanding of past and future changes in this region in the context of global warming. Here, we obtained a complete permafrost core from the Mohe Basin in the Greater Khingan Mountains in Northeast China. We reconstructed the hydrology and ecological evolution of the permafrost region since the Late Pleistocene (30 cal. ka BP) using organic geochemical evidence (including TOC, TN, C/N ratio, stable isotopes δ^13^C_org_ and δ^15^N) from a permafrost core, as well as AMS^14^C dating. The results indicate that the environment in the permafrost region in the Mohe Basin closely aligned with the glacial, deglaciation, and interglacial. Between the Last Mega‐Interstadial and the Last Glacial Maximum (30–19 cal. ka BP), the surface runoff gradually decreased, primary productivity was low, the wetlands shrank until they disappeared, and a grassland ecosystem formed. During the Last Deglaciation (19–11.5 cal. ka BP), although the organic matter content fluctuated, the surface runoff and primary productivity increased, and the catchment area of the watershed expanded, leading to redevelopment of the wetlands, and a coniferous‐dominated mixed forest wetland ecosystem formed. In the Early to Middle Holocene (11.5–5.8 cal. ka BP), primary productivity and surface runoff increased further, gradual wetland expansion reached the maximum extent, and a mixed coniferous‐broadleaved forest wetland ecosystem formed. During the Middle to Late Holocene (since 5.8 cal. ka BP), primary productivity and surface runoff decreased, and the wetland water level declined, initiating peatland development and ultimately a coniferous forest swamp ecosystem formed. We believe that the ice volume in the Northern Hemisphere and the East Asian summer monsoon alternately controlled the regional hydrology and ecological evolution during different periods.

## Graphical abstract

Continuous hydrology and ecological evolution records of permafrost regions in Northeast China since the late Pleistocene (30 cal. ka BP) are obtained. Responses of hydrology and ecosystem dynamics to the climate cycle on the millennial scale and regional environmental effects related to permafrost. The ice volume in the Northern Hemisphere and East Asian summer monsoon alternately controlled the hydrology and ecological evolution.

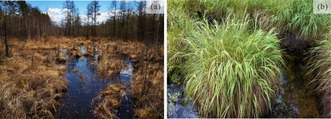

## 1 Introduction

In the context of intensifying global climate warming, past global changes (PAGES) and their ecological effects have become a focus of attention among governments around the world and have been included in the International Geosphere‐Biosphere Programme (IGBP). Among them, hydrological and ecological changes in mid‐high latitude regions are an important topic (IPCC 2022). Most of the land in mid‐high latitude regions in the Northern Hemisphere (north of 48° N) is covered by permafrost (Zhang et al. 1999). Permafrost is a direct product of cold climates, and climate promotes the development of the ecological environment in this permafrost region (Bonan et al. 1992; Gałka et al. 2018).

As a groundwater aquiclude of soil, permafrost not only regulates the regional hydrological balance through dynamic changes in its upper seasonal thawing layer (active layer) but also preserves a large amount of solid groundwater (underground ice). The presence of permafrost increases the flow of snowmelt and rainfall in the watershed (Zhao et al. 2015). In addition, the water‐blocking effect and low‐temperature conditions of permafrost lead to the upward migration of water, salts, and nutrients in the soil, ensuring the vitality of the permafrost active layer soil. This plays a crucial role in maintaining water circulation and ecological stability and is also the reason for the widespread distribution of lakes, wetlands, and swamps in permafrost regions (Luthin and Guymon 1974; Soja et al. 2007). The coupling of the surface hydrological conditions and ecosystems in permafrost regions obstructs the energy exchange between the permafrost and atmosphere, providing protection for the continuation of the underlying permafrost and forming a special type of ecologically protective permafrost (Shur and Jorgenson 2007). Additionally, it indirectly influences the regional microclimate (Jin et al. 2008; Chang et al. 2011). Generally, climate‐hydrology, hydrology‐ecology, and ecology‐permafrost systems have interactive relationships in permafrost regions (Liu et al. 2023). The hydrology and ecosystem exhibit sensitive responses to changes in the climate and permafrost environment (Zhao et al. 2016; Tikhonravova 2023).

Since the Late Pleistocene (30 cal. ka BP), the world has experienced several notable alternations between cold and warm periods, including a series of millennial‐scale climate oscillations such as the Heinrich (H) events, Dansgaard‐Oeschger cycles, and Younger Dryas (YD) event (Clark et al. 2009; Zhang et al. 2022). Frequent alternation between cold and dry and warm and wet climate conditions inevitably leads to multiple episodes of expansion and contraction of permafrost (Zhao et al. 2014). These changes not only influence hydrological processes in permafrost regions, such as groundwater levels, surface runoff, and regional water cycling, but may also drive transitions in ecosystem responses across different ecosystem types, including grasslands, forests, and wetlands (Camill and Clark 2000). Therefore, understanding the hydrology and ecological evolution processes in permafrost regions is essential for assessing the past and future climate and environmental development.

The permafrost in Northeast China is mainly distributed in the Greater Khingan Mountains (GKM), which are located north of 46° N. Controlled by the latitude gradient of the surface temperature, from north to south, discontinuous permafrost gradually transforms into isolated patches/sporadic permafrost. While during the Last Glacial Maximum (LGM), the southern boundary of the permafrost reached the Bohai Bay at 41°–42° N (Jin et al. 2019). Previous studies on the hydrology and ecological records have been widely conducted in Northeast China, mainly in areas such as the GKM (Wu and Liu 2012; Wen et al. 2017; Chen et al. 2021; Sun et al. 2023), Changbai Mountains (Hong et al. 2001; Liu et al. 2008; Parplies et al. 2008; Zhou et al. 2010; Stebich et al. 2015), Inner Mongolia Plateau (Wen et al. 2010; Zhang et al. 2022), Songnen Plain, and Sanjiang Plain (Wang et al. 2015; Liu et al. 2017; Zhang, Yao, et al. 2021). Previous studies have shown that the regional climate pattern is correlated with the dominance of the East Asian summer monsoon (EASM), and moisture and vegetation are sensitive to changes in the EASM. However, these studies mainly focused on non‐permafrost regions and alpine permafrost regions in central‐southern Northeast China, while proxy records for the concentrated distribution of permafrost in Northeast China are lacking, and the relationship between the hydrology and ecological evolution and the permafrost remains unclear.

Here, we present organic geochemical evidence from a complete permafrost core collected in the Mohe Basin of Northeast China. A chronological framework was constructed based on accelerator mass spectroscopy (AMS) ^14^C dating, and linear correlation analysis was applied. By comparing paleoecological records from the surrounding region with global climate records, we aim to achieve the following objectives: (1) to reconstruct the continuous hydrology and ecological evolutionary processes of the permafrost region since the Late Pleistocene (30 cal. ka BP); (2) to identify the possible forcing of regional hydrology and ecological evolution during different periods. This study not only provides reliable data to support the understanding of hydrology and ecological evolution in permafrost regions, but also provides critical insights for addressing global climate change and conserving vulnerable ecosystems.

## 2 Geographic Setting

**FIGURE 1:**
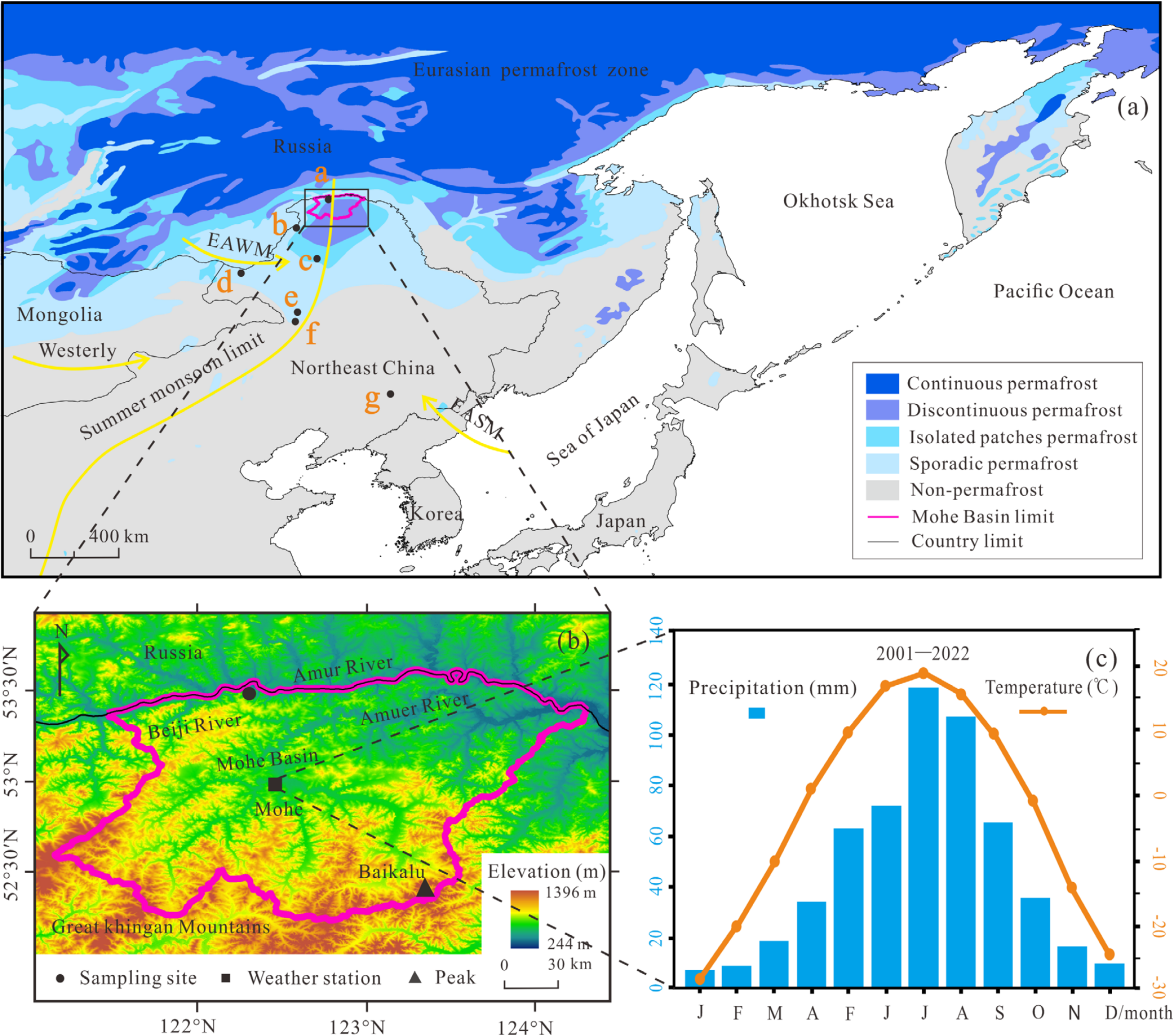
(a) Location of the Mohe Basin in the Eurasian permafrost region, and other sites discussed in this study. a. Mohe Basin (BJC–3 core); b. Wuma ice wedge (Tong 1993); c. Yitulihe ice core (Yang and Jin 2011); d. Hulun Lake (Zhang et al. 2022); e. Moon Lake (Chen et al. 2021); f. Tofengling Lake (Sun et al. 2023); g. Erlongwan Lake (Liu et al. 2008). The summer monsoon limit from Han et al. (2019). (b) The regional elevation of the Mohe Basin. (c) Meteorological records from the Mohe weather station (data from http://data.cma.cn).

The Mohe Basin (52°10′–53°33′ N, 121°07′–124°20′ E, 450 m average a.s.l.) (Figure 1b) is located in the northernmost part of China, that is, in the northern part of the GKM in northeast China. Structurally, the Mohe Basin is part of the Erguna Block in the Mongolia‐Okhotsk orogenic belt. During the Late Jurassic, separation processes resulted in the formation of a faulted basin (Li et al. 2017). The northern side of the Mohe Basin is bounded by the Amur River and is connected to the Upper Amur Basin in Russia. The terrain in the Mohe Basin is hilly and undulating due to fluvial erosion caused by the Amur River and its tributaries, and numerous wetlands have formed in low‐lying areas. The Mohe Basin's periphery is the main ridge of the GKM, and the highest peak is Baikalu at 1396 m a.s.l.

The MoHe Basin represents the northern margin of the EASM region and is characterized by a cold‐temperate, continental, monsoonal climate. Winters are cold and dry under the influence of the cold polar airmass, and summers are warm and humid under the influence of the northwestern Pacific Ocean airmass. It has a mean annual temperature (MAT) of −4.9°C (Figure 1c), monthly mean temperatures of −29.7°C in January and 19.8°C in July, and a historical winter temperature extreme of −53°C. The mean annual precipitation (MAP) is 468.9 mm, and more than 80% of the precipitation is concentrated from June to September. The Mohe Basin is the protruding southward part of the Eurasian permafrost zone. The surface freezes for more than 8 months of the year, and the mean annual ground temperature (MAGT) remains below 0°C. The permafrost distribution area is 1.84 × 10^4^ km^2^, and the average frozen layer is ~60 m thick, making it a typically discontinuous permafrost region (Figure 1a).

**FIGURE 2:**
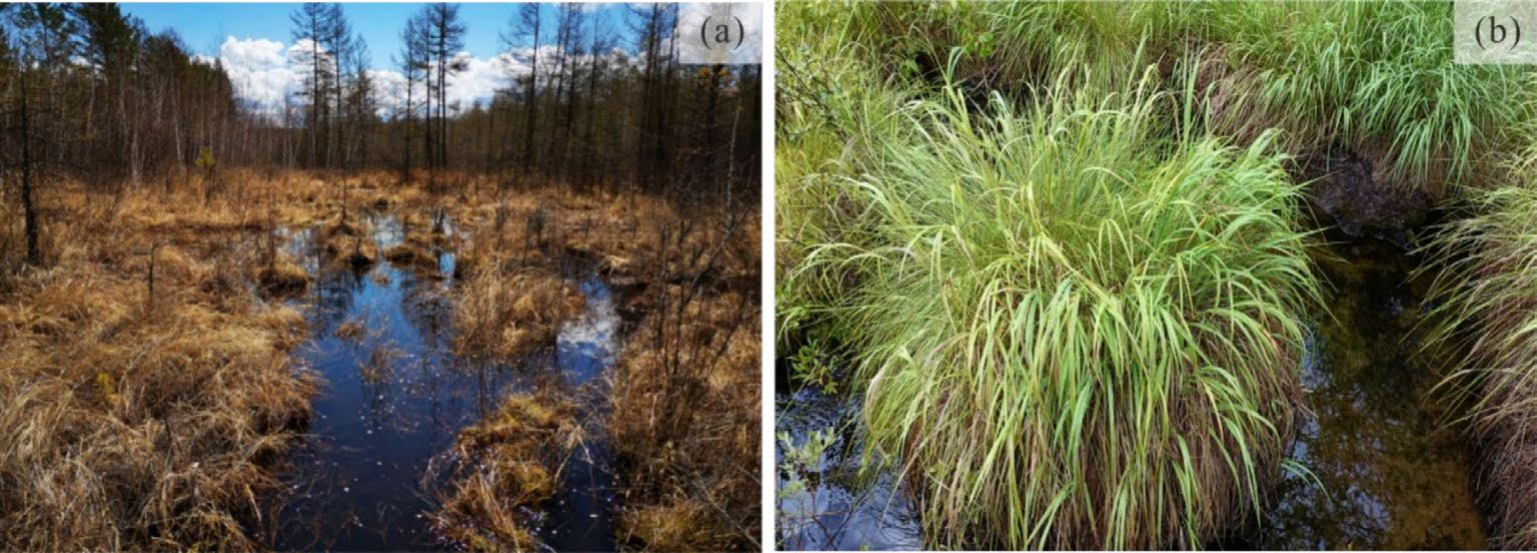
Landscapes of the Mohe Basin. (a) Current status of permafrost wetland. (b) Typical plant *Carex meyeriana* (Cyperaceae).

The regional vegetation and soil distributions have distinct altitudinal zonality. (1) In the low‐lying areas in the basin (< 300 m a.s.l.), because excessively wet, mesotrophic or oligotrophic permafrost wetlands have formed, the soil mainly consists of peat‐swamp soil, and the vegetation includes *Carex meyeriana*, *Polypodiode snipponica*, *Epilobium hirsutum* , and Bryophytes (Figure 2). (2) In the hilly and low mountainous areas in the basin (300–650 m a.s.l.), the surface is slightly wet, the soil is mainly dark coniferous forest, and the vegetation includes *Larix gmelinii* , *Pinus sylvestris* , *Betula platyphylla* , *Alnus hirsuta*, and *Corylus heterophylla* , as well as small amounts of *Rhododendron dauricum* and *Betula fruticosa*. (3) In the mountainous areas on the periphery of the basin (> 650 m a.s.l.), the surface is relatively dry, the soil is mainly dark coniferous forest soil or gravelly soil, and the vegetation includes *Picea koraiensis*, *L. gmelinii* , and *Pinus pumila* , as well as *Artemisia* and Poaceae (Sun et al. 2011).

## 3 Materials and Methods

### 3.1 Sampling of Core BJC–3

A 742 cm long permafrost core was collected in September 2017 (BJC**–**3 core, 53°28′17″ N, 122°19′47″ E, 298 m a.s.l.). An XY–2000 Russia geological drill was used to drill through the permafrost layer and down to the bedrock. The core was split, photographed, and described on site. The core recovery rate was calculated to be 92.5%.

**FIGURE 3:**
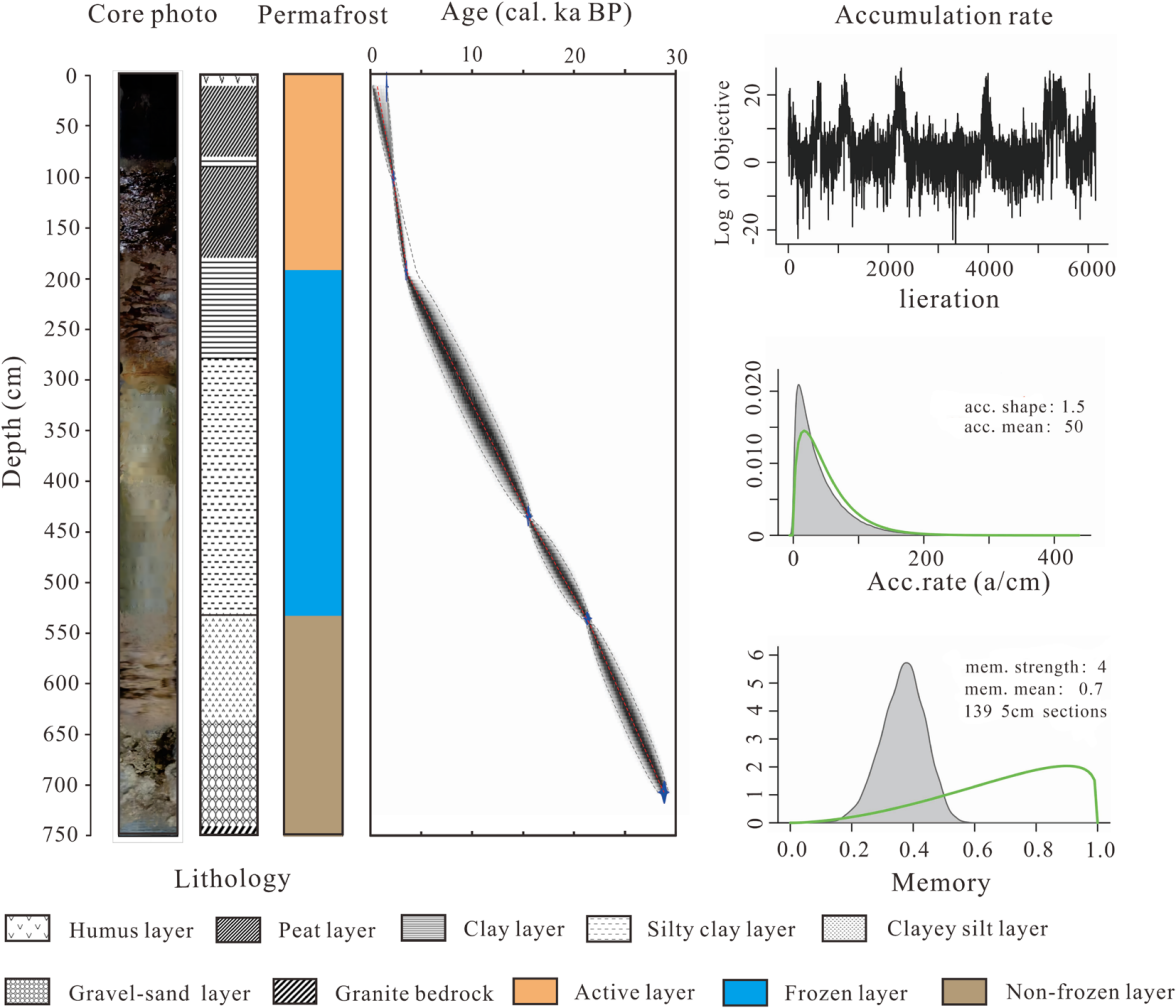
Core photo, lithology, permafrost structure, and age‐depth model of BJC–3 core.

The lithology of core BJC–3 consists of a humus layer (0–15 cm), a peat layer (15–70 cm), a clay layer (70–80 cm), a peat layer (80–170 cm), a clay layer (170–270 cm), a silty clay layer (270–530 cm), a clayey silt layer (530–640 cm), and a gravel‐sand layer (640–742 cm). The core comprises eight distinct layers, predominantly composed of wetland sedimentary facies. The base is granite bedrock formed by magmatic intrusion, which exhibits an unconformable contact with the overlying Quaternary deposits. The permafrost structure consists of three layers from top to bottom: an active layer (0–180 cm), a permafrost layer (180–530 cm), and a non‐frozen layer (530–742 cm) (Figure 3).

Because the samples consisted of a peat‐ice mixture, the core was allowed to partially thaw to obtain sufficient material. Sediment samples were then systematically sliced at 5 cm intervals from the top downward using a stainless steel knife. A total of 148 samples were collected and freeze‐dried in the laboratory.

### 3.2 AMS^14^C Dating

The high accuracy of ^14^C dating in determining the age of sediments younger than 50 000 years makes it a commonly used method for dating permafrost cores (Mokhova et al. 2009; Jones et al. 2012; Estop‐Aragonés et al. 2020). Due to the high ice content of core BJC–3, we selected layers with high organic material contents for sampling. Six samples, including peat, organic sediment, and mud, were selected for AMS^14^C dating. The analysis was performed at Beta Analytic Inc. in the USA.

Tree rings curve calibrated correction based on the Intal 20 Northern Hemisphere terrestrial standard dataset was conducted using the Calib 8.1 software (with BP corresponding to 1950 ce), and the AMS^14^C correction ages had a high confidence interval (95%) of 2*σ* ages and median ages (Reimer et al. 2013). The age‐depth model was constructed using the “Bacon” piecewise linear accumulation model in R (Blaauw and Christen 2011), with sedimentation rate interpolation applied to estimate ages at 1 cm resolution. The resulting chronology was used to create a piecewise linear cumulative age‐depth relationship graph (Figure 2).

The AMS^14^C ages for core BJC–3 were placed in chronological order from young to old (Table 1), and the AMS^14^C age curve shows an overall linear trend (Figure 3). We estimate that the lowermost age of core BJC–3 is ~30 cal. ka BP, and the mean sedimentation rate of the core was calculated to be ~0.25 mm/a.

**TABLE 1:** AMS^14^C dating of BJC–3 core in the Mohe Basin.

Depth (cm)	Permafrost structure	Dated material	Conventional age (year BP)	2*σ*‐range age (year BP)	Median age (cal year. BP)
20	Active layer	Bulk peat	1440 ± 30	1297–1373	1331
110	Active layer	Bulk peat	2070 ± 30	1973–2117	2031
190	Active layer	Organic sediment	3110 ± 30	3234–3391	3328
430	Frozen layer	Mud	12,831 ± 30	15,186–15,506	15,315
530	Frozen layer	Mud	17,450 ± 50	20,885–21,295	21,031
710	Non‐frozen layer	Organic sediment	24,530 ± 100	28,642–28,996	28,778

### 3.3 Organic Geochemistry Analysis

Organic geochemistry is a widely used proxy that can reflect the ecological patterns of lakes and wetlands. Organic matter buried in core sediments, including total organic carbon (TOC), total nitrogen (TN), total organic carbon/total nitrogen (C/N) ratio, stable isotope δ^13^C_org_ and δ^15^N values, can reveal the regional past primary productivity, nutrient cycling, surface moisture, and the correlation between ecological pattern and the climate/environment.

Visible impurities were removed from the fresh sediment samples, and then the remaining sample was dried at 60°C for 24 h. First, the samples were ground into powder finer than > 200 mesh. Then, 10% HCl was added to 2 g of sample and left to stand for 8 h to remove the carbonate in the sediment. After this, the samples were washed in centrifuge tubes with deionized water to neutral pH and were freeze dried. The sample was ground again to < 150 mesh and dried at 45°C for 24 h (Parplies et al. 2008). The TOC and TN were analyzed using a Jena multi N/C 3100 analyzer from Germany. The standard deviations of the TOC and TN values are less than 1%, and the TOC and TN values are expressed as content percentages (%). The C/N ratios were calculated as TOC/TN (mass percent). The δ^13^C_org_ and δ^15^N were analyzed using a Thermo Fisher MAT–253 stable isotope mass spectrometer from the USA. The precisions were routinely checked by running the urea isotopic working standard (No. IVA33802174) after every 10 sample measurements. The standard deviation of the δ^13^C values is less than 0.15‰, and the standard deviation of the δ^15^N values is less than 0.1‰.

### 3.4 Statistical Analyses

Linear correlation analysis was used to quantify the statistical association between two organic geochemistry indicators, and further to explore their potential relationships with trends in environmental parameters. The linear regression equation is expressed as *y* = *bx* + *a*, where *y* represents the dependent variable, *x* denotes the independent variable, *b* is the slope coefficient quantifying the rate of change, and *a* is the *y*‐intercept indicating the baseline value. The best‐fit line is determined by least‐squares minimization to represent the relationship between variables. In linear regression, the closer the correlation coefficient *R*‐value is to 1 or −1, the higher the degree of correlation. The probability *p*‐value is employed to indicate the significance of the correlation between variables. Specifically, when *p* < 0.01 (99% confidence level), it suggests an extremely significant linear relationship between the two variables, while *p* < 0.05 (95% confidence level) implies a significant linear relationship. All computations were conducted in Origin 2021.

## 4 Results and Discussion

### 4.1 Organic Geochemistry Characteristics

**FIGURE 4:**
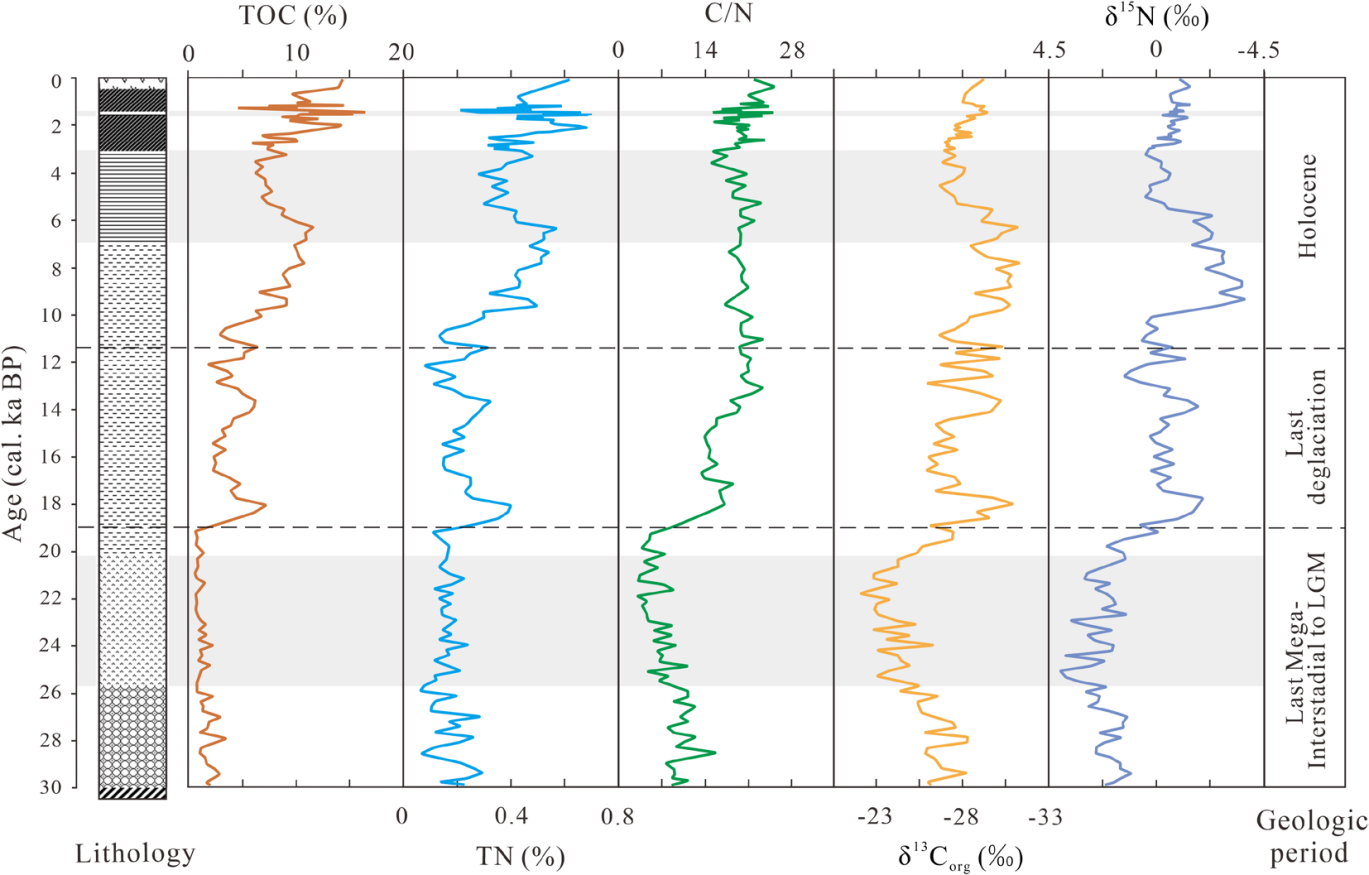
Lithology, TOC, TN, C/N ratio, δ^13^C_org_, and δ^15^N of BJC–3 core. The gray strip represent lithologic sequence, black dashed line represents the boundaries between different geologic periods.

The TOC, TN, C/N ratio, and stable isotope δ^13^C_org_ and δ^15^N values are presented in Figure 4. The average TOC content is 5.56%, with a range of 0.57%–16.8%. The maximum value occurs at 1.6 cal. ka BP, and the minimum value occurs at 19 cal. ka BP. The average TN content is 0.32%, with a range of 0.06%–0.74%. The maximum value occurs at 1.7 cal. ka BP, and the minimum value occurs at 25.8 cal. ka BP. The average C/N ratio is 15.56, with a range of 3.26–25.58. The maximum value occurs at 1.6 cal. ka BP, and the minimum value occurs at 19 cal. ka BP. The average δ^13^C_org_ value is −26.78‰, with a range of −31.01‰ to −21.12‰. The maximum value occurs at 21.7 cal. ka BP, and the minimum value occurs at 7.8 cal. ka BP. The average δ^15^N value is 0.47‰, with a range of −3.11‰ to 4.03‰. The maximum value occurs at 24.9 cal. ka BP, and the minimum value occurs at 9.3 cal. ka BP.

**FIGURE 5:**
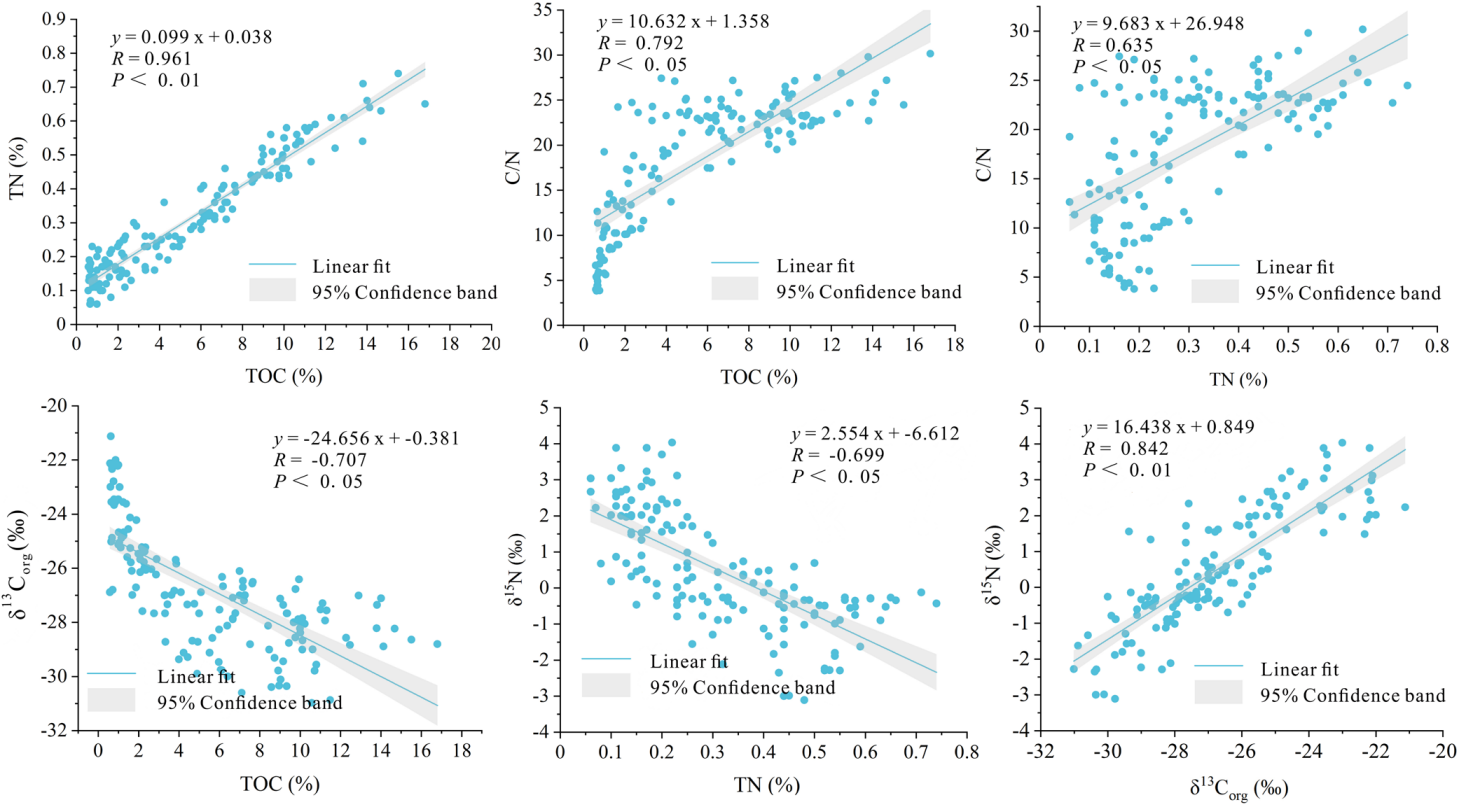
Scatter plots showing the correlations between TOC and TN, TOC and C/N, TN and C/N, TOC and δ^13^C_org_, TN and δ^15^N, δ^13^C_org_ and δ^15^N.

In core BJC–3, the TOC content, TN content, and C/N ratio exhibit gradually decreasing trends from top to bottom, while the δ^13^C_org_ and δ^15^N values exhibit gradually increasing trends. Some linear correlation results among various indicators are shown in Figure 5. TOC and TN (*R* = 0.961, *p* < 0.01), δ^13^C_org_ and δ^15^N (*R* = 0.842, *p* < 0.01) showed a highly significant positive correlation; TOC and C/N (*R* = 0.792, *p* < 0.05), TN and C/N (*R* = 0.635, *p* < 0.05) showed a significant positive correlation; in contrast, TOC and δ^13^C_org_ (*R* = −0.707, *p* < 0.05), TN and δ^15^N (*R* = −0.699, *p* < 0.05) showed a significant negative correlation. These results indicate significant changes in various indicators among different geological periods in the Mohe Basin (Figure 4). Thus, the changes in organic matter of the BJC–3 core reflected the characteristics of glacial, deglaciation, and interglacial periods. We divided the ecological environment evolution into three stages to investigate these changes.

### 4.2 Source and Significance of Organic Matter

Changes in organic geochemistry indicators are linked to the abundance of organic matter in sediments, which is composed of biological proteins, fatty acids, carbohydrates, etc. (Rao et al. 2008). Sedimentary organic matter is generally considered to be a regional marker, and its source can reflect the regional hydrology and ecological pattern (Talbot and Johannessen 1992). The source of the organic matter in wetland sediments is similar to that in lakes, mainly from the slow decomposition of plant residue, which is related to endogenous and exogenous plant sedimentation (Meyers 1994). The Mohe Basin is not a completely enclosed region. Although the input of organic matter may include contributions from seasonal wind transport and animal migration, notably, the basin's topographic confinement (elevation difference > 400 m relative to adjacent mountains) results in endogenous organic matter being predominantly sourced from wetland vegetation, while exogenous organic matter is mainly supplied through fluvial redistribution of terrestrial plants from the watershed.

The TOC and TN are comprehensive reflections of the regional primary productivity, and the C/N ratio can indicate the composition of the regional vegetation sources (Brahney et al. 2008). Generally, C/N ratios of < 10 indicate that the wetland plants are primarily composed of endogenous low plants with high‐protein contents, while ratios > 20 indicate that the wetland plants are composed of exogenous terrestrial vascular plants with high‐cellulose contents (Krishnamurthy et al. 1986). Furthermore, the C/N ratio can also reflect changes in wetland water levels and the supply ability of the regional surface runoff, indirectly indicating the climate and environmental conditions (Meyers 1990). The Mohe Basin's average TOC (5.56%) and TN (0.32%) contents indicate regional primary productivity comparable to surrounding lakes (Parplies et al. 2008; Xiao et al. 2008). The Mohe Basin exhibits an average C/N ratio of 15.59, indicating that the organic matter primarily originates from a mixed input of endogenous plants and exogenous plants. Concurrently, the variation ranges of TOC (0.57%–16.8%), TN (0.06%–0.74%), and C/N ratios (3.26–25.58) show substantial variability, suggesting that regional primary productivity and vegetation source composition were influenced by climatic and environmental changes during different periods.

The ranges of the δ^13^C_org_ and δ^15^N values also reflect the types of exogenous/terrestrial and endogenous/aquatic plants in the region (Kohn 2010). Terrestrial plants can be classified into C_3_, C_4_, and crassulacean acid metabolism (CAM) plants based on their carbon fixation via photosynthesis and primary production method (Talbot and Johannessen 1992). C_3_ plants are generally suited to atmospheric environments with higher CO_2_ concentrations. All trees, most shrubs, and herbs of cryophilic and hygrophilous are C_3_ plants. The average δ^13^C_org_ value of C_3_ plants is −27‰, with a range of −30‰ to −24‰. C_4_ plants are mainly herbs and are mainly distributed in low‐latitude and low‐altitude areas with high temperatures. The average δ^13^C_org_ value of C_4_ plants is −14‰, with a range of −19‰ to −9‰. CAM plants mainly grow in barren desert environments. The average δ^13^C_org_ value of CAM plants is −17‰, with a range of −30‰ to −10‰ (Meyers 1994). The natural δ^15^N_Air_ value is ~0‰. Since terrestrial plants (particularly C_3_ plants) can directly assimilate atmospheric N_2_ during nitrogen fixation, their δ^15^N values are typically low, ~1‰ (Bustamante et al. 2004). The Mohe Basin shows variation ranges of δ^13^C_org_ (−31.01‰ to −24.12‰) and δ^15^N (−3.11‰ to 4.03‰) indicating C_3_ plant‐dominated vegetation. This is consistent with the inference that regional vegetation in mid‐high latitude regions should not contain C_4_ or CAM plants (Ehleringer et al. 1997).

Aquatic plants can generally be classified into emergent plants, phytoplankton, and submerged plants. Emergent plants directly utilize atmospheric CO_2_ as the carbon source for photosynthesis, and their δ^13^C_org_ values range from −30‰ to −24‰, which are close to those of C_3_ plants. If phytoplankton utilize atmospheric CO_2_ as the carbon source for photosynthesis, their δ^13^C_org_ value range is close to those of emergent plants and C_3_ plants. However, if they utilize ${\mathrm{HCO}}_{3}^{-}$ in water as the carbon source, their δ^13^C_org_ value range will be higher. Submerged plants utilize ${\mathrm{HCO}}_{3}^{-}$ in water as the carbon source for photosynthesis, and their δ^13^C_org_ values range from −20‰ to −12‰ (Meyers and Lallier‐Vergès 1999). Moreover, aquatic plants also absorb dissolved inorganic nitrogen (DIN) from water for photosynthesis, resulting in relatively high δ^15^N values of > 2‰ (Peterson and Howarth 1987). The relatively low ground temperature of wetlands within the Mohe basin, coupled with the dense surface vegetation, results in the underwater light in the wetlands being poor, making regional submerged plants very rare (Zhou 1991). Therefore, the regional vegetation is mainly within the range of terrestrial C_3_ plants, emergent plants, and phytoplankton. The main sources of the organic matter were various contributions from endogenous/aquatic plants and exogenous/terrestrial plants, and the reason for the changes in the composition of δ^13^C_org_ and δ^15^N is the response to climate and environmental changes.

According to the results of linear correlation analysis, TOC and δ^13^C_org_ (*R* = −0.707, *p* < 0.05), TN and δ^15^N (*R* = −0.699, *p* < 0.05) showed a significant negative correlation. This confirms that Wang et al. (2002) proposed a decreasing trend in δ^13^C_org_ and δ^15^N values with increasing moisture and temperature in northern China, whereas TOC, TN, and C/N ratio showed the opposite pattern, increasing with increasing moisture and temperature. In summary, a higher contribution of exogenous organic matter typically reflects greater regional primary productivity and surface runoff in a warm and wet climate. A higher contribution of endogenous organic matter typically reflects a larger presence of wetland aquatic plants, lower moisture, and a cold and dry climate. The changes in the compositions of the organic matter in the Mohe Basin since 30 cal. ka BP not only reflect the regional ecological pattern but also the implicit changes in the regional hydrology. The changes in the organic matter better reflect the hydrology and ecological evolution in the Mohe Basin and its peripheral mountains.

### 4.3 Hydrology and Ecological Evolution Processes

By analyzing the response relationships of organic matter to climate cycles/oscillations (including the H1–3 events, Local Last Glacial Maximum (LLGM), Bølling–Allerød (B–A) period, YD, Holocene Megathermal Period (HMP), and Neoglacial) and permafrost dynamics in the Mohe Basin, we reconstructed the hydrology and ecological evolution processes in the permafrost region of Northeast China during different geologic periods.

#### 4.3.1 Last Mega‐Interstadial (LMI) to LGM (30–19 cal. ka BP)

**FIGURE 6:**
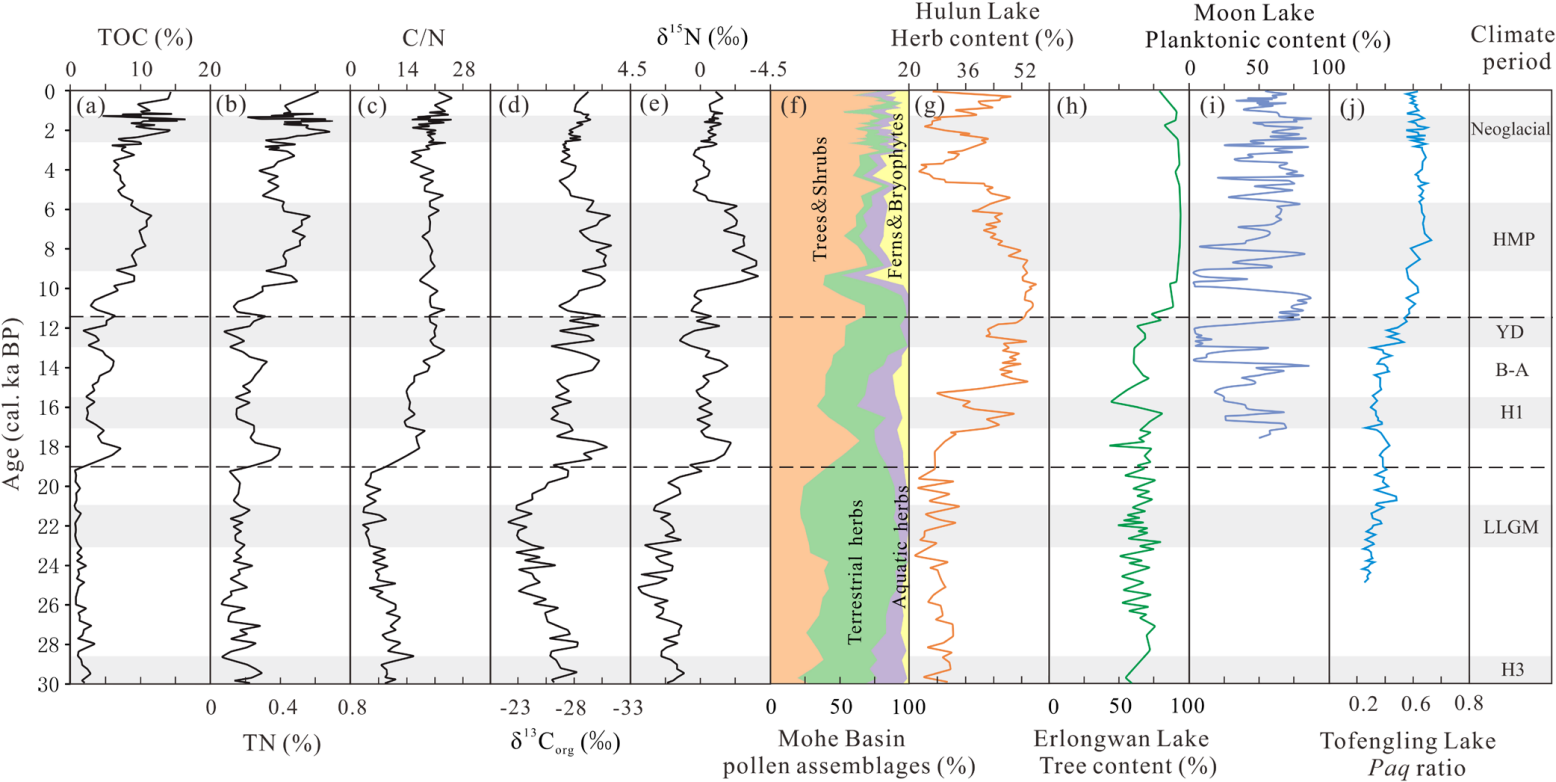
(a–e) Comparison of organic matter record from BJC–3 core. (f) Pollen of the Mohe Basin (Liu et al. 2024). (g) Pollen of Hulun Lake (Zhang et al. 2022). (h) Pollen of Erlongwan Lake (Liu et al. 2008). (i) Diatom of Moon Lake (Chen et al. 2021). (j) N‐Alkanes of Tofengling Lake (Sun et al. 2023). The black dashed line represents the division of intervals between glacial, deglaciation and interglacial.

The sedimentation during this period mainly corresponds to the gravel‐sand layer and clayey silt layer, which indicate that the sedimentary environment was mainly dominated by freeze–thaw weathered material and sandstorm sedimentation. These changes are characterized by decreased TOC and TN contents coupled with a lower C/N ratio (Figure 6a–c). These changes indicate a decrease in the organic matter input, reduced regional surface runoff, decreased vegetation coverage, and low primary productivity, with lower plants being dominant. The δ^13^C_org_ and δ^15^N showed an increasing trend (Figure 6d,e), indicating a transition of the organic matter from exogenous C_3_ plants to endogenous phytoplankton, and leading to the wetlands shrinking until they disappeared. Until the harshest climatic period in the Local Last Glacial Maximum (LLGM, 23–21 cal. ka BP), the regional organic matter input decreased to the greatest extent, indicating a further decrease in surface vegetation and a possible deterioration of the basin's ecological environment. These changes reflect that the region has undergone a long‐term climate cooling process since the H3 event in the Late Pleistocene, exacerbating the effects on the ecological environment.

This conclusion is also supported by the surrounding paleoecological records. The dunes began to develop in the western part of Northeast China during the period (Yang and Yue 2013). Pollen records from Hulun Lake indicate that the environment has deteriorated, sand dunes have gradually replaced the grassland, and the grasslands have expanded eastward, constituting the main landscape of the eastern part of Northeast China (Zhang et al. 2022) (Figure 6g). Erlongwan Lake records reveal a decrease in tree pollen and an increase in herbaceous plants (Liu et al. 2008) (Figure 6h). In the Mohe Basin, terrestrial herbs (e.g., *Artemisia*, Chenopodiaceae, and Poaceae) rapidly expanded and became the absolute dominant species, and the abundance of trees was also very low (Liu et al. 2024) (Figure 6f). Similarly, the aquatic proxy (*paq*) ratio of Tofengling Lake sediments indicates that the area of the lake surface rapidly decreased, which suggests a low‐biomass environment with low vegetation coverage (Sun et al. 2023) (Figure 6j).

It can be inferred that during this period, driven by the cold and dry climate and expansion of the permafrost area, the surface was a frozen and dry environment with reduced regional vegetation cover. The low organic matter content suggests a grassland ecosystem formed in this region, rather than the previously assumed landscape pattern dominated entirely by coniferous forests (Li et al. 2019).

#### 4.3.2 Last Deglaciation (19–11.5 cal. ka BP)

The sedimentation during this period corresponds to the silty clay layer. These sediments reflect an improved sedimentary environment, manifested by rapid increases in TOC and TN contents, along with elevated C/N ratios. (Figure 6a–c). These changes indicate an increase in the organic matter input. Expansion of the watershed led to enhanced surface runoff, increased vegetation coverage, recovery of the regional primary productivity, and an increase in the higher‐plants abundance. The δ^13^C_org_ and δ^15^N were significantly reduced (Figure 6d,e), indicating an increase in the exogenous organic matter input from C_3_ plants, while the endogenous organic matter was from a mixture of emergent plants and phytoplankton. This suggests that the wetlands began to develop again. However, the fluctuations in organic matter reflect the unstable ecological environment in the Mohe Basin during the Last Deglaciation (Figure 6a–e). During the H1 event (17–15.6 cal. ka BP), the decrease in organic matter resulted from greater surface runoff and wetland expansion, which in turn facilitated the spread of aquatic herbs, ferns, and bryophytes within the basin. This situation improved until the Bølling–Allerød (B–A) interspace (15.6–13 cal. ka BP), during which an increase in organic matter indicated the restoration of regional vegetation cover and primary productivity, and enhanced surface runoff contributed to rising wetland water levels. During the YD event (13–11.5 cal. ka BP) organic matter showed a fluctuating decline. Unlike during the H1 event, during the YD, the decrease in the C/N ratio was not significant (average 20.58), suggesting lush growth of regional C_3_ plants while wetland areas may have shrunk.

These hydrology and ecosystem fluctuations are also recorded in regional paleoecological archives. Pollen records show that during the Last Deglaciation, the dominant vegetation shifted from *Pinus* to *Betula* in the Mohe Basin. The expansion of thermophilic broadleaved forests (e.g., *Ulmus*, *Carya*, and *Tilia*) coincided with a decline in aquatic plants (e.g., Cyperaceae and Onagraceae) (Liu et al. 2024) (Figure 6f). In Hulun Lake, regional land cover experienced repeated alternations between dunes and grassland (Zhang et al. 2022) (Figure 6g), while Erlongwan Lake records document multiple pronounced shifts in arboreal pollen assemblages (Liu et al. 2008) (Figure 6h). The reconstructed hydrology records from Moon Lake and Tuofengling Lake also indicate that lake levels fluctuated significantly due to climatic variability during this period (Chen et al. 2021; Sun et al. 2023) (Figure 6i,j).

Based on these changes, it can be inferred that although there were fluctuations of organic matter in the Mohe Basin during the Last Deglaciation, the regional hydrology and ecological conditions underwent significant transformation. The thawing of permafrost and mountain snowpack increased regional moisture, reactivated wetland systems, and triggered vegetation succession, leading to the establishment of a coniferous‐dominated mixed forest wetland ecosystem.

#### 4.3.3 Holocene (11.5 cal. ka BP to Present)

The sedimentation during this period corresponds to the clay layer and peat layer, with a decrease in sediment sand and gravel content, indicating that the sedimentary environment became more stable. After entering the Holocene, the TOC and TN contents gradually increased to their maximum levels, while the C/N ratio remained stable at a high value. Concurrently, the δ^13^C_org_ and δ^15^N values gradually decreased to their minimum levels (Figure 6a–e). This indicates that the regional primary productivity and surface runoff increased further. Vegetation coverage reaches its highest level, the organic matter input from exogenous C_3_ plants became dominant, and the endogenous organic matter was primarily sourced from emergent plants. These findings suggest an expansion in both the wetland water level and area.

However, the accumulation of organic matter progressed slowly during the Early Holocene (11.5–9 cal. ka BP) (Figure 6a–e). Research shows that although temperatures increased in Northeast China during this interval, moisture conditions remained persistently dry, which retained characteristics of the YD period. This aridity inhibited rapid development of the regional hydrology and ecosystem (Yu et al. 2008; Wu and Liu 2012; Stebich et al. 2015). A significant shift occurred with the onset of the HMP (9–5.8 cal. ka BP) (Figure 6a–e). In the Early to Middle Holocene, the sand‐drift activity in the western part of Northeast China almost stagnated, and paleosols replaced the sand deposits. The entirety of Northeast China experienced a relatively calm climate period. The pollen record from the Mohe Basin shows that the regional trees expanded and became dominant, and the proportion of thermophilic broadleaved forests reached the maximum, thus replacing the aquatic herbs and ferns (Liu et al. 2024) (Figure 6f). Lake‐level reconstructions from Moon Lake and Tuofengling Lake indicate synchronous water‐level increases corresponding to peak regional humidity conditions (Chen et al. 2021; Sun et al. 2023) (Figure 6i,j). This reflects that the Mohe Basin had a warm and wet climate during the HMP, and a mixed coniferous‐broadleaved forest wetland ecosystem formed. At 5.8 cal. ka BP, there was a continuous decrease in organic matter input (Figure 6a–e), which resulted in a decrease in regional primary productivity and surface runoff, a reduction in vegetation cover, a decrease in organic matter input from exogenous C_3_ plants, and an increased contribution from endogenous phytoplankton. These changes suggest a decline in wetland water levels and areal extent, fostering environmental conditions favorable for the onset of regional peatland formation. Especially since 2.9 cal. ka BP, peatlands have transitioned from eutrophic to mesotrophic or oligotrophic types (Xia 1996), accompanied by increased variability in sediment organic matter abundance.

Numerous pollen records for Northeast China recorded a *Pinus* expansion event at 5–6 cal. ka BP (Gao et al. 2022), which is generally consistent with the timing of the climate cooling in Northern China (Li et al. 2016). This reflects the widespread change in climate and marks the end of the HMP. Thus, in the Late Holocene, climate was conducive to the expansion of coniferous forest and wetlands, which provided a material basis for peat development and ultimately led to the formation of a coniferous forest wetland ecosystem. This differs from the gradual trend toward a warm and dry climate in the northern part of Northeast China (Liu et al. 2008; Ma et al. 2021). These findings support the conclusion of Zheng et al. (2018) that the climate became colder and wetter during this period, and this climate characteristic was also the main reason for the vigorous development of peat in the Mohe Basin (Xing et al. 2015).

### 4.4 Possible Forcing for Hydrology and Ecological Evolution

Research indicates that the hydrology and ecological evolution in Northeast China has been closely linked to climate dynamics (Zhang et al. 2020). Temperature fluctuations are driven by orbitally induced variations in summer solar radiation modulated in the mid‐high latitudes of the Northern Hemisphere (Wen et al. 2010), while precipitation patterns are predominantly controlled by the EASM and surrounding ocean–atmosphere interactions (An 2000). Furthermore, climate change has also influenced the region by affecting atmospheric CO_2_ concentrations, the westerly jet stream, and ice volume in the Northern Hemisphere (including the Eurasian ice sheet, sea ice, and permafrost) (Horiuchi et al. 2000; Zhou et al. 2016).

#### 4.4.1 LMI to LGM (30–19 cal. ka BP)

**FIGURE 7:**
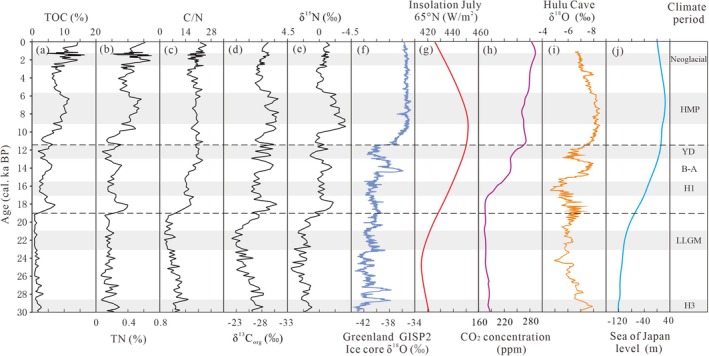
(a–e) Comparison of organic matter results from BJC–3 core with potential forcings. (f) Ice core δ^18^O of Greenland GISP2 (Stuiver and Grootes 2000). (g) Insolation July at 65° N (Berger 1978). (h) Ice core CO_2_ of Antarctica EPICA Dome C (Ahn et al. 2004). (i) Speleothem δ^18^O of Hulu Cave (Wang et al. 2001). (j) Sea‐level reconstruction of Japan (Spratt and Lisiecki 2016).

After the end of the middle of the last glacial warm period (i.e., LMI), the low organic matter content in the Mohe Basin indicates reduced primary productivity, decreased regional surface runoff, and the wetlands shrank until they disappeared, resulting in the formation of a barren grassland ecosystem. Global paleoclimate records show that during this period, the continuous decrease in Northern Hemisphere summer insolation (Berger 1978) (Figure 7g) marked a synchronous regional cooling, with estimated global terrestrial surface temperatures (TST) decreasing by 6°C–8°C (Osman et al. 2021; Seltzer et al. 2021). Greenland ice core δ^18^O record demonstrates the expansion of ice volume in the Northern Hemisphere (Stuiver and Grootes 2000) (Figure 7f), while the Eurasian ice sheet began to redevelop at 25 cal. ka BP (Siegert et al. 2001), and permafrost extent reached its maximum (Ehlers and Gibbard 2007; Vandenberghe et al. 2014).

The deterioration of the ecological environment in the Mohe Basin is mainly attributed to the expansion of the Eurasian ice sheet, sea ice in the Sea of Japan, and regional permafrost. Ice sheet expansion diminishes land vegetation cover, elevating surface albedo and intensifying the meridional temperature gradient, while also causing cooling and drying across the entire Asian continent under the control of the Mongolian–Siberian High. Moreover, the atmospheric CO_2_ concentration was only ~60% of the current value (Ahn et al. 2004) (Figure 7h), further suppressing surface vegetation growth and leading to sparse vegetation cover and a continuously deteriorating ecological environment in the Mohe Basin. Simultaneously, the expansion of ice volume promoted the formation of sea ice in the Sea of Japan and a sea‐level drop (Spratt and Lisiecki 2016) (Figure 7j), hindering the northward advance of the EASM monsoon and thereby reducing moisture exchange between the ocean and land. This process intensified the aridity in the Mohe Basin, and its occurrence period was highly consistent with the weakened phase of the EASM indicated by the Hulu Cave δ^18^O record (Wang et al. 2001) (Figure 7i). In addition, the cold climate also facilitated the permafrost expansion. The frozen and arid surface environment inevitably hindered the regional water vapor cycle, leading to reduced wetland recharge and their shrinkage until they disappeared. Regional trees were forced to migrate southward or become extinct. The region became more suitable for the expansion of cold and drought‐resistant herbs with shallow roots (Ma et al. 2018). Also, the expansion of the permafrost inhibited plant growth by restricting nutrient availability, water absorption, and space for root activities in the frozen soil, thereby further decreasing the transport of organic matter to the wetlands. Consequently, these factors contributed to the formation of a barren grassland ecosystem. The expansion of ice volume in the Northern Hemisphere during this period was the main driving force behind the evolution of regional hydrology and ecosystems.

#### 4.4.2 Last Deglaciation (19–11.5 cal. ka BP)

Although there were fluctuations of organic matter in the Mohe Basin during the Last Deglaciation, the surface runoff and primary productivity increased; wetlands were restored, hydrology and ecological conditions were effectively improved, establishing a coniferous‐dominated mixed forest wetland ecosystem. Compared with global paleoclimate records, it was found that during this period, as the summer insolation in the Northern Hemisphere increased, the global temperature rapidly responded during 20–18 cal. ka BP (Clark et al. 2009), which coincided with the timing of the retreat of the Eurasian ice sheet and permafrost (Svendsen et al. 2004). The higher temperatures resulted in the production of meltwater from the permafrost and mountain snowpack, which led to an increase in atmospheric CO_2_ emissions (Figure 7h). This meltwater not only accelerated the rapid humidification process of the Mohe Basin, facilitating water circulation and vegetation growth, but also contributed to a sea‐level rise in the Sea of Japan (Spratt and Lisiecki 2016) (Figure 7j), which facilitated hydrothermal exchange between the land and sea. The intensified summer monsoon transported moisture from the Sea of Japan to the Mohe Basin, resulting in an increase in the effective precipitation (Wang et al. 2001) (Figure 7i). Thus, the regional hydrology and ecological conditions improved. Sedimentological evidence from the MH Basin supports this interpretation: the mud layer at 500–460 cm in core BJC–3 contains a large amount of yellow silt, which may have originated from regional flooding during this period, indicating a sudden increase in moisture.

Additionally, based‐pollen climate reconstructions suggest a potential climatic teleconnection between Northeast China and the North Atlantic (Stebich et al. 2009). Due to the H3 and H2 events during the LGM cooling period, the response of the ecological environment in the Mohe Basin was not significant. During the H1 event during the Last Deglaciation, the periodic drift of ice rafts in the North Atlantic intensified the pressure gradient between the high‐ and low‐latitude regions, which was a driving force of the reduced surface temperature and precipitation in the Northern Hemisphere. Although the EASM significantly weakened during the H1 event, some records from Northeast China indicate that the effective humidity increased. This is mainly attributed to the sea‐level rise in the Sea of Japan (Spratt and Lisiecki 2016) (Figure 7j), the intensification of the Northwest Pacific High, which induced the exchange of moisture between land and sea (Dyke 2004; Zhang et al. 2018), and the southward shift of the westerlies, which maintained a partial regional moisture balance (Bond et al. 1992; Denton et al. 2010). These factors collectively promoted the restoration of wetlands and aquatic plants in the Mohe Basin. These hydroclimatic conditions persisted until the early stage of the Bølling–Allerød (B–A) warming interval, at which time the EASM began to strengthen, leading to the development of the regional ecological environment. The Atlantic Meridional Overturning Circulation (AMOC) during the YD event weakened the humidity and temperature transport from the low latitude to high latitude regions (Broecker et al. 1988), resulting in a corresponding weakening of the EASM (Wang et al. 2001) (Figure 7i). As a result, the increase in the ice volume in the Northern Hemisphere regained control of the regional climate and environment. Based on the reconstruction of the YD period using the GKM Wuma ice wedge, the MAT was 6°C lower than the current value, and the precipitation was only 20% of the current value (Tong 1993). These conditions severely constrained the development of wetlands and aquatic plants in the Mohe Basin. Moreover, the climate lag caused by the residual ice sheet and regional permafrost led to the continuation of this drought condition into the Early Holocene. The δ^18^O record of the Greenland ice core (Stuiver and Grootes 2000) and the δ^18^O record of Hulu Cave (Wang et al. 2001) both support this conclusion (Figure 7g,i).

Collectively, during the Last Deglaciation, the rapid humidification in the early‐middle stage significantly promoted hydrology and ecological development in the Mohe Basin. Most of the Northeast China region had entered a wetland development period (Zhou et al. 2010; Zhang et al. 2020). In comparison, the humidification process likely began earliest in the Mohe Basin. In the late stage, wetland areas progressively contracted in response to diminished meltwater supply, intensified evapotranspiration, and broadleaf forest expansion, reflecting deteriorating moisture availability and rising temperatures. Therefore, the driving forces behind the regional hydrology and ecological evolution were complex, resulting from the interaction between the Northern Hemisphere ice volume and the EASM, which increased the instability of the regional ecological environment changes.

#### 4.4.3 Holocene (11.5 cal. ka BP to Present)

With the advent of the Holocene, the organic matter content in the Mohe Basin further increased, indicating a gradual improvement in regional primary productivity and surface runoff, along with gradual wetland expansion to its maximum extent, forming a mixed coniferous‐broadleaved forest wetland ecosystem. Based on multiple indicators records (e.g., pollen, diatom, *n*‐alkanes, and organic geochemistry), the ecological environment in Northeast China has responded sensitively to the EASM since the Holocene (Parplies et al. 2008; Wen et al. 2017; Han et al. 2023; Sun et al. 2023). In the Early to Middle Holocene (9–5.8 cal. ka BP), the increase in the summer insolation in the Northern Hemisphere triggered a series of chain reactions, leading to the complete melting of the Eurasian ice sheet at ~10 cal. ka BP (Svendsen et al. 2004), a continuous increase in atmospheric CO_2_ emissions (Ahn et al. 2004) (Figure 7h), and a continuous sea‐level rise in the Sea of Japan (Spratt and Lisiecki 2016) (Figure 7j). The organic matter in the Mohe Basin also increased, and the advancement of the strengthened EASM system was the driving force of the development of regional forests and wetlands during the period. Under the warm climate and more precipitation, the establishment of forests increased the mineralization of the soil in the watershed, leading to increased surface runoff and the input of a large amount of organic matter into the wetlands (Zhang, Wünnemann, et al. 2021). Meanwhile, due to the aquiclude effect of the incomplete thawed permafrost, the surface water could not infiltrate, which caused the soil moisture to increase in the active layer. This condition facilitated gleyization and the development of wetlands, and the water level and area of wetlands expanded to the maximum values. Moreover, the expansion of the wetlands also affected the growth of plants and provided activity space for plant root growth and soil nutrient accumulation. Thus, these conditions provided the necessary foundation for the development of forests and wetlands in the Mohe Basin.

Subsequently, at 5.8 cal. ka BP, the summer insolation in the Northern Hemisphere and the EASM began to weaken synchronously (Berger 1978; Wang et al. 2001) (Figure 7g,i), and the climate experienced cooling and drying, resulting in the regional wetland water level declining and coniferous forest development. Since the Neoglacial period (2.5 cal. ka BP), the Yitulihe ice wedge in the GKM has recorded multiple expansions of regional permafrost (Yang and Jin 2011). A cold climate promoted a decrease in the decomposition rate of regional plant residues, while the vigorous development of permafrost peatlands not only reduced surface water evaporation but has also maintained the balance between the climate and permafrost, promoting the current coexistence of wetlands/peatlands and permafrost (Ran et al. 2021). Therefore, these changes reflect the sensitive response of the ecological environment to EASM in the Mohe Basin since the Holocene.

In summary, the hydrology and ecological evolution in the Mohe Basin has been influenced by multiple factors since 30 cal. ka BP, but its fundamental driving force can be attributed to the alternating influences of ice volume in the Northern Hemisphere caused by the EASM changes.

## 5 Conclusions

This study provides insights into the hydrology and ecological evolution in the permafrost region in Northeast China, as well as their potential forcing mechanisms, based on TOC, TN, C/N ratio, δ^13^C_org_, and δ^15^N records from core BJC–3 from the Mohe Basin of GKM, can be summarized as follows:

The hydrology and ecological evolution reflect the glacial, deglaciation, and interglacial changes in the Mohe Basin. Between the LMI and LGM, between the Last Mega‐Interstadial and the Last Glacial Maximum (30–19 cal. ka BP), the surface runoff gradually decreased; primary productivity was low, the wetlands shrank until they disappeared, and a grassland ecosystem formed. During the Last Deglaciation, although the organic matter content fluctuated, the surface runoff and primary productivity increased, and the catchment area of the watershed expanded, leading to the redevelopment of the wetlands, and a coniferous‐dominated mixed forest wetland ecosystem formed. In the Early to Middle Holocene (11.5–5.8 cal. ka BP), primary productivity and surface runoff increased further, gradual wetland expansion reached the maximum extent, and a mixed coniferous‐broadleaved forest wetland ecosystem formed. During the Middle to Late Holocene (since 5.8 cal. ka BP), primary productivity and surface runoff decreased, and the wetland water level declined, initiating peatland development, and ultimately a coniferous forest swamp ecosystem formed.

We conclude that the ice volume in the Northern Hemisphere and the EASM alternately controlled the regional hydrology and ecological evolution during different periods. Between the LMI and LGM, the increase in the ice volume in the Northern Hemisphere (including the Eurasian ice sheet, sea ice, and permafrost) was the driving force of the regional hydrology and ecological evolution. During the Last Deglaciation, the driving forces of the regional hydrology and ecological evolution were complex due to the interplay between effects of the ice volume in the Northern Hemisphere and the EASM. This increased the instability of the climate and environmental change. Since the Holocene, the regional hydrology and ecological evolution have responded sensitively to the fluctuations of the EASM. Our results suggest that the ecological environmental evolution in Northeast China may not have been entirely dominated by the EASM climate pattern, and regional environmental effects caused by the increase in the ice volume in the Northern Hemisphere cannot be ignored.

## Author Contributions

**Rui Liu:** data curation (lead), methodology (lead), writing – original draft (lead). **Lin Zhao:** conceptualization (lead). **Xiaodong Wu:** writing – review and editing (equal). **Xiaofeng Cheng:** methodology (equal), visualization (equal). **Boxiong Zhang:** investigation (equal). **Jianxiang He:** investigation (equal). **Dongyu Yang:** visualization (equal). **Shuying Zang:** funding acquisition (lead), project administration (lead).

## Conflicts of Interest

The authors declare no conflicts of interest.

## Supporting information


**Data S1:** ece371935‐sup‐0001‐Supinfo.xlsx.

## Data Availability

Data available in article Supporting Information.
